# Molecular detection of *Enterocytozoon bieneusi* in alpacas (*Vicugna pacos*) in Xinjiang, China

**DOI:** 10.1051/parasite/2019031

**Published:** 2019-05-28

**Authors:** Qiyuan Zhang, Haiyan Wang, Aiyun Zhao, Wei Zhao, Zilin Wei, Zhiguo Li, Meng Qi

**Affiliations:** 1 College of Animal Science, Tarim University Alar Xinjiang 843300 PR China; 2 Experimental and Research Center, Henan University of Animal Husbandry and Economy Zhengzhou Henan 450046 PR China; 3 Department of Pathogenic Biology, Hainan Medical University Haikou Hainan 571100 PR China

**Keywords:** *E. bieneusi*, alpacas, genotype, ITS, zoonotic

## Abstract

*Enterocytozoon bieneusi*, an obligate intracellular pathogen, can infect a wide variety of hosts. This study aimed to determine the prevalence and molecular characteristics of *E. bieneusi* in alpacas (*Vicugna pacos*) in China. A total of 185 alpaca fecal samples were collected from five herds in Tacheng, Wensu, Hejing, Qinghe, and Nilka counties in Xinjiang Uygur Autonomous Region. *Enterocytozoon bieneusi* was detected by nested PCR of the internal transcribed spacer (ITS) region. Twenty-eight fecal samples (15.1%, 28/185) were positive for *E. bieneusi*, with the highest prevalence in alpacas from Qinghe (42.9%, 15/35). Four *E. bieneusi* genotypes were identified, which included two known (P and ALP3) and two novel (ALP7 and ALP8) genotypes. Genotype ALP3 was the dominant genotype (57.1%, 16/28), followed by genotypes P (32.1%, 9/28), ALP7 (7.1%, 2/28), and ALP8 (2.6%, 1/28). Phylogenetic analysis revealed that three genotypes (P, ALP7, and ALP3) clustered into group 1, whereas genotype ALP8 clustered into group 8. This is the first report of *E. bieneusi* infection and genetic diversity in alpacas from Xinjiang, China.

## Abstract (fr)

*Enterocytozoon bieneusi*, un agent pathogène intracellulaire obligatoire, peut infecter une grande variété d’hôtes. Cette étude visait à déterminer la prévalence et les caractéristiques moléculaires d’*E. bieneusi* chez les alpagas (*Vicugna pacos*) en Chine. Au total, 185 échantillons de selles d’alpagas ont été prélevés dans cinq troupeaux des comtés de Tacheng, Wensu, Hejing, Qinghe et Nilka, dans la région autonome ouïgoure du Xinjiang. *Enterocytozoon bieneusi* a été détecté par PCR nichée de la région de l’espaceur interne transcrit (ITS). Vingt-huit échantillons de selles (15,1 %, 28/185) étaient positifs pour *E. bieneusi*, la prévalence étant la plus élevée chez les alpagas de Qinghe (42,9 %, 15/35). Quatre génotypes d’*E. bieneusi* ont été identifiés, dont deux génotypes connus (P et ALP3) et deux nouveaux (ALP7 et ALP8). Le génotype ALP3 était le génotype dominant (57,1 %, 16/28), suivi des génotypes P (32,1 %, 9/28), ALP7 (7,1 %, 2/28) et ALP8 (2,6 %, 1/28). Une analyse phylogénétique a révélé que 3 génotypes (P, ALP7 et ALP3) appartenaient au groupe 1, tandis que le génotype ALP8 appartenait au groupe 8. Il s’agit du premier rapport sur l’infection à *E. bieneusi* et sa diversité génétique chez les alpagas du Xinjiang en Chine.

## Introduction

*Enterocytozoon bieneusi*, an unicellular fungi, has a broad host range (humans, livestock, companion animals and wildlife) and has even been detected in environmental water samples [2, 12, 13]. On the basis of sequence analysis of the ribosomal internal transcribed spacer (ITS) region, at least 340 *E. bieneusi* ITS genotypes have been reported in humans and animals [8, 14, 18]. Phylogenetic analysis revealed that these ITS genotype sequences were clustered into at least 10 large groups (groups 1–9 and a so-called outlier in dogs). Among them, group 1 contains most of the genotypes found in humans, while the remaining groups mostly include host-adapted genotypes found in specific animals, such as ruminants, nonhuman primates, and dogs [12–14, 16, 18].

Alpacas (*Vicugna pacos*), which originated in South America, were imported into China from Australia in 2002. Today, alpacas in China are mainly raised for meat and wool, and for the sightseeing industry. However, limited information is available about the prevalence and genetic characteristics of *E. bieneusi* in alpacas, except for three reports from Peruvian, Australian, and Chinese zoos, where genotypes ALP1–6, BEB6, CHALT1, D, J, P, and Type IV were obtained [3–6]. China has an estimated total herd size of nearly 4000 alpacas, with the largest number of alpacas (over 500 animals) in Xinjiang Uygur Autonomous Region (hereafter referred to as Xinjiang), northwestern China [19]. Compared with other livestock, such as cattle and horses, nothing is known about *E. bieneusi* infection in alpacas from Xinjiang. Therefore, the aim of this study was to investigate *E. bieneusi* prevalence in alpacas from Xinjiang, and to assess the genetic diversity of *E. bieneusi* isolates by ITS sequence analysis.

## Methods

### Sample collection

**Figure 1:**
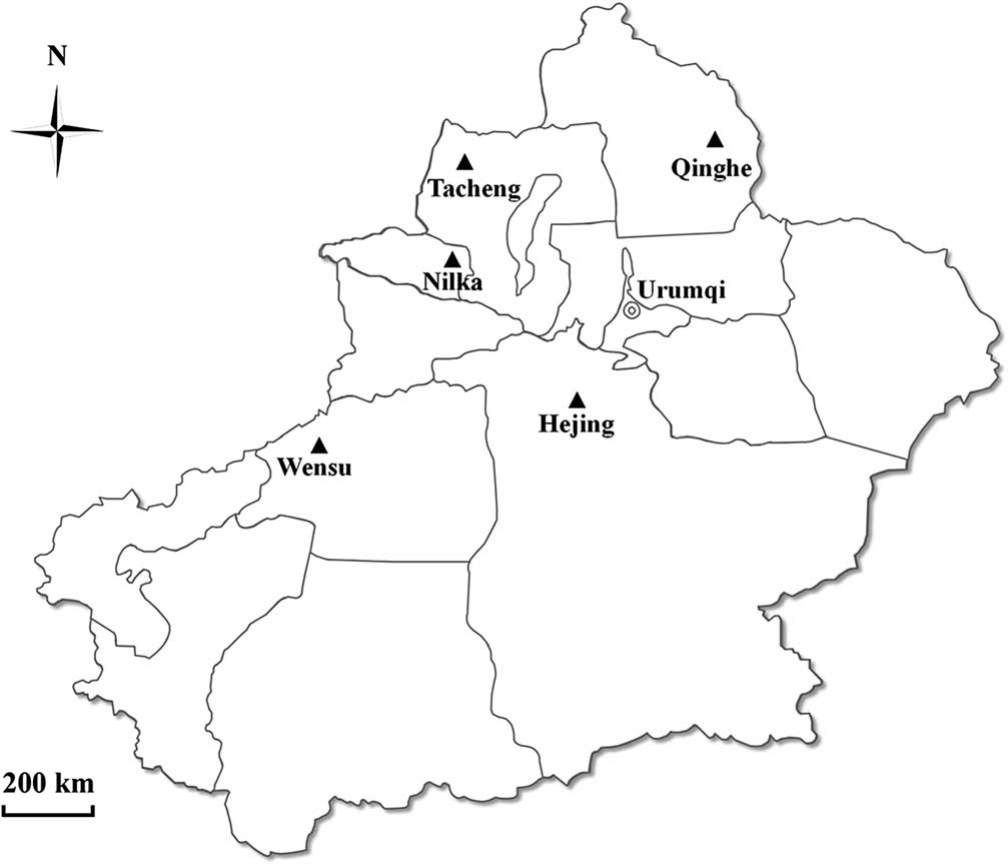
Specific locations from which samples were collected in this study. ▲ Study locations.

From August 2016 to March 2017, a total of 185 fresh fecal samples were collected from five herds of alpacas in Tacheng (46°21′ N–41°14′ N, 82°41′ E–83°41′ E,), Wensu (79°28′ E–81°30′ E, 40°52′ N–42°15′ N), Hejing (82°28′ E–87°52′ E, 42°06′ N–43°33′ N), Qinghe (89°47′ E–91°04′ E, 45°00′ N–47°20′ N), and Nilka (81°85′ E–84°58′ E, 43°25′ N–44°17′ N) counties in Xinjiang, China (Fig. 1). Each herd contained 27–380 animals; the collected samples accounted for approximately 20%–50% of alpacas in each herd. All of these animals were fed hay and had shelter at night, but also freely grazed in a fenced pasture during the day. The alpacas are segregated in fences of enclosure and sampling was carried out in the enclosure. Fresh fecal samples (20–30 g) were collected using sterile gloves and were placed into clean labeled plastic bags immediately after animal defecation. No diarrhea was observed during sampling. A total of 185 individual alpaca fresh fecal samples were collected. All the samples were transported to our laboratory in a cooler with ice packs within 48 h. The fecal samples were stored at 4 °C and DNA was extracted within 1 week.

### DNA extraction and PCR amplification

An aliquot (3–5 g) of each fecal sample was diluted with distilled water through a wire mesh sieve (250 μm pore size) into a 10 mL centrifuge tube and centrifuged at 5000 ×*g* for 3 min; then, the supernatant was removed. Genomic DNA was extracted from approximately 200 mg fecal samples using the E.Z.N.A.^®^ Stool DNA Kit (Omega Biotek Inc., Norcross, GA, USA), according to the manufacturer’s instructions. Then, 200 μL of extracted DNA from each sample were transferred to Eppendorf tubes and stored at −20 °C until PCR amplification.

*Enterocytozoon bieneusi* was detected by nested PCR of the ITS region, as previously described [15]. A 25 μL PCR mixture was used for PCR amplification, and contained 12.5 μL 2× EasyTaq PCR SuperMix (TransGene Biotech Co. Ltd., Beijing, China), 10.9 μL deionized water, 0.3 μM of each primer, 1 μL genomic DNA for the primary PCR, and 1 μL primary amplification product for the secondary PCR. The secondary PCR products were examined by electrophoresis in a 1.5% agarose gel and stained with GelRed™ (Biotium Inc., Hayward, CA, USA).

### Sequencing and phylogenetic analysis

The positive secondary PCR products obtained for the ITS region (392 bp) were sent to a commercial company (GENEWIZ, Suzhou, China) for bidirectional sequencing. The nucleotide sequences were compared with reference sequences downloaded from the National Center for Biotechnology Information (https://www.ncbi.nlm.nih.gov/) using Clustal X 2.1 (http://www.clustal.org/) to determine the *E. bieneusi* genotypes.

Bayesian inference (BI) and the Monte Carlo Markov Chain (MCMC) method were used to construct phylogenetic trees in MrBayes v 3.2.6 (http://mrbayes.sourceforge.net/). FigTree v 1.4.4 (http://tree.bio.ed.ac.uk/software/figtree/) was used to visualize and edit the maximum clade credibility tree generated by these analyses. Posterior probability values were estimated based on 1,000,000 generations with four simultaneous tree building chains, with trees being saved every 100th generation. A 50% majority rule consensus tree for each analysis was constructed based on the final 75% of trees generated by BI. The ITS nucleotide sequences of *E. bieneusi* obtained in this study were submitted to GenBank under the accession numbers MH998003–MH998006.

## Results and discussion

In the present study, of the 185 fecal samples tested for *E. bieneusi* by nested PCR, 28 (15.1%) were positive. Three out of the five alpaca herds were positive, and the highest infection rate (42.9%, 15/35) was detected in animals from Qinghe (Table 1). The overall infection rate of *E. bieneusi* (15.1%) in this study was lower than that reported in alpacas in the highlands of Peru (51.6%) [3], and higher than that in Australia (9.9%) [4]. Currently, *E. bieneusi* has been isolated from humans, nonhuman primates, pigs, cattle, sheep, yaks, deer, cats and dogs, chickens, rodents, and snakes, as well as urban wastewater in China [18]. Xinjiang has an abundance of herbivore livestock (including cattle, sheep, goats, yaks, Bactrian camels, horses, and deer), and is a major producer and consumer of livestock products in China. The infection rate of *E. bieneusi* (15.1%) in alpacas in this study was similar to that reported in dairy calves (16.5%), and lower than that reported in grazing horses (30.9%), and Bactrian camels (30.0%) in Xinjiang [9–11]. These differences may be due to different animal groups and sample size. To our knowledge, this is the first molecular investigation of *E. bieneusi* in alpacas from Xinjiang.

**Table 1:** *Enterocytozoon bieneusi* prevalence in alpacas from Xinjiang, northwestern China.

Location	No. examined	No. positive (%)	Genotype (no.)
Tacheng	18	0	**–**
Wensu	100	12 (12.0)	ALP3 (1), ALP7 (2), P (9)
Hejing	20	1 (5.0)	ALP8 (1)
Qinghe	35	15 (42.9)	ALP3 (15)
Nilka	12	0	–
Total	185	28 (15.1)	ALP3 (16), ALP7 (2), ALP8 (1), P (9)

Among the 28 *E. bieneusi* ITS nucleotide sequences, four genotypes (two known genotypes, P and ALP3, and two novel genotypes, ALP7 and ALP8) were identified in this study. Genotype ALP3 (*n* = 16) was the dominant genotype, followed by P (*n* = 9), ALP7 (*n* = 2), and ALP8 (*n* = 1) (Table 1). Among the three herds that were positive for *E. bieneusi*, genotype ALP3 (*n* = 15) was detected in Qinghe, genotype ALP8 (*n* = 1) in Hejing, and genotypes ALP3, ALP7, and P in Wensu (Table 1). Until now, molecular investigation of *E. bieneusi* in alpacas has been limited to two studies from farmed alpacas in Peru and Australia [3, 4], and two studies from captive alpacas in Chinese zoos [5, 6]. A total of 14 *E. bieneusi* genotypes (P, ALP1–8, Type IV, D, BEB6, J, and CHALTI) have been identified in alpacas worldwide [3–6], and genotype ALP1 was dominant in Peru and Australia [3, 4] (Table 2). Interestingly, only two known genotypes (ALP3 and P) and two novel genotypes (ALP7 and ALP8) were identified in this study, with genotype ALP3 (57.1%, 16/28) being the most frequent genotype, followed by genotype P (42.8%, 9/28). Genotype ALP3 was only detected in two farmed alpaca samples in Peru and Australia [3, 4], whereas genotype P was first identified in captive llamas (*Lama glama*) from Munich Zoo [1], and then later detected in fecal samples of alpacas from Peru and Australia [3, 4]. The differences in the predominance of *E. bieneusi* genotypes in different areas indicate that *E. bieneusi* infection in alpacas may exhibit regional differences. The lack of investigations into alpaca infection by *E. bieneusi* indicates that more studies should be undertaken to compare differences between areas.

**Table 2:** Summary of known *Enterocytozoon bieneusi* genotype distributions in alpacas worldwide.

Region	Collection site	No. of samples	No. positive (%)	Genotype (no.)	Reference
China	Zoo	4	3 (75.0)	CHALT1 (1), J (2)	[5]
China	Zoo	1	1 (100)	BEB6 (1)	[6]
Peru	Farms	126	65 (51.6)	ALP1 (48), ALP2 (1), ALP3 (1), ALP4 (1), ALP5 (1), ALP6 (1), BEB6 (1), D (2), P (5), Type IV (4)	[3]
Australia	Farms	81	8 (9.9)	ALP1 (5), ALP3 (1), P (2)	[4]
China	Farms	185	28 (15.1)	ALP3 (16), ALP7 (2), ALP8 (1), P (9)	This study
Total				ALP1 (53), ALP2 (1), ALP3 (18), ALP4 (1), ALP5 (1), ALP6 (1), ALP7 (2), ALP8 (1), BEB6 (2), CHALT1 (1), D (2), J (2), P (16), Type IV (4)	

In previous studies, various dominant genotypes of *E. bieneusi* infection were found in different animals in Xinjiang, such as genotypes J and I in dairy calves [9], genotypes EbpC and EpbA in grazing horses [11], and genotype CAM1 in Bactrian camels [10]. In this study, genotypes ALP3 and P were the predominant genotype; these results indicate that animal-derived *E. bieneusi* in Xinjiang may have host adaptation. Clearly, this hypothesis needs to be verified by further epidemiological surveys.

**Figure 2:**
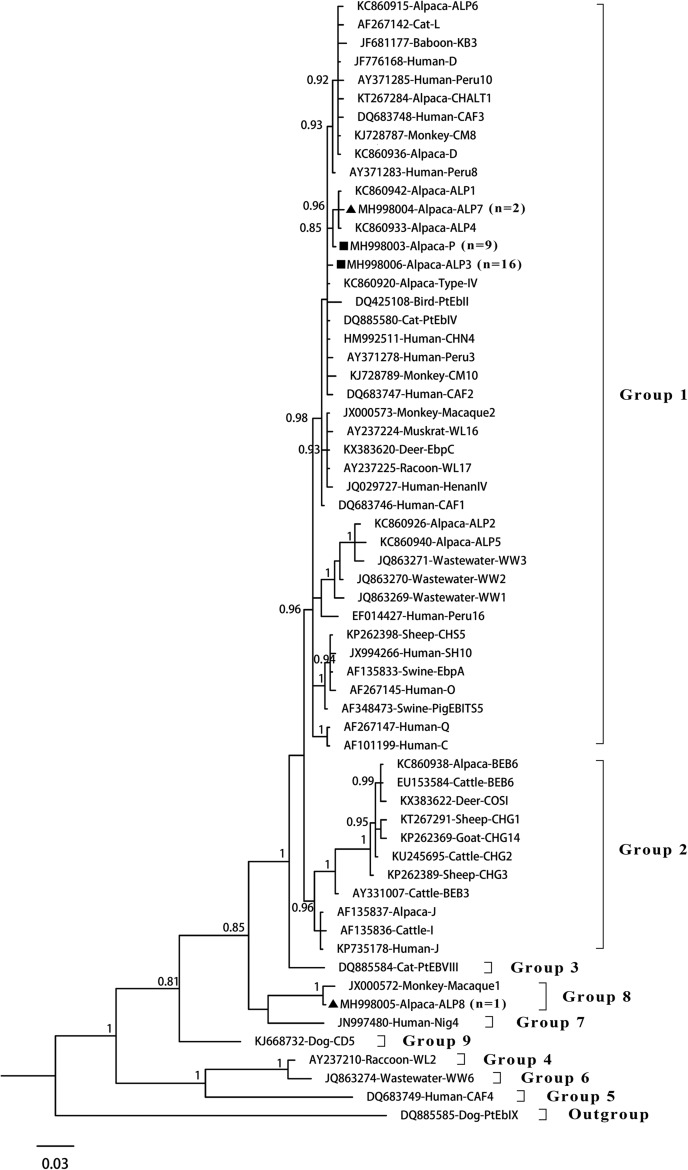
Bayesian phylogenetic analysis of *Enterocytozoon bieneusi* ITS sequences. Statistically significant posterior probabilities are indicated at branches. Sample names include GenBank accession number followed by host and then genotype designation. The *E. bieneusi* genotype PtEbIX (DQ85585) from dogs was used as outgroup. Known and novel genotypes identified in this study are indicated by squares and triangles, respectively.

The phylogenetic analysis based on ITS sequencing revealed that genotypes ALP3, ALP7, and P belonged to group 1, whereas genotype ALP8 belonged to group 8 (Fig. 2). Among the 14 *E. bieneusi* genotypes identified in alpacas worldwide to date [3–6], 11 genotypes (D, Type IV, CHALT1, P, and ALP1–7) clustered into group 1, of which genotypes D and Type IV have been detected in human samples [7]. Genotypes J and BEB6 clustered into group 2, which was composed of genotypes that were mostly obtained from *E. bieneusi* in ruminants [17, 20]. Genotype ALP8 clustered into group 8, which also contained some genotypes from nonhuman primates and Bactrian camels, such as genotypes KB-5, Macaque1, CAM1, CAM2, and CAM4 [10, 18]. Therefore, future studies should evaluate the molecular epidemiology of *E. bieneusi* in other hosts to elucidate the transmission dynamics of the identified genotypes.

## Conclusions

To our knowledge, this is the first report on *E. bieneusi* infection and genetic diversity in alpacas from Xinjiang. Our results indicate that *E. bieneusi* infection is prevalent among alpacas in this region. Moreover, phylogenetic analysis based on ITS sequencing revealed that most *E. bieneusi* isolated from these alpacas belonged to group 1.

## Competing interests

The authors declare that they have no competing interests.
